# A unique cysteine-rich zinc finger domain present in a majority of class II ribonucleotide reductases mediates catalytic turnover

**DOI:** 10.1074/jbc.M117.806331

**Published:** 2017-10-02

**Authors:** Christoph Loderer, Venkateswara Rao Jonna, Mikael Crona, Inna Rozman Grinberg, Margareta Sahlin, Anders Hofer, Daniel Lundin, Britt-Marie Sjöberg

**Affiliations:** From the ‡ Department of Biochemistry and Biophysics, Stockholm University, SE-106 91 Stockholm, Sweden and; the § Department of Medical Biochemistry, Umeå University, SE-901 87 Umeå, Sweden

**Keywords:** metal ion–protein interaction, oligomerization, oxidation-reduction (redox), phylogenetics, ribonucleotide reductase, thioredoxin

## Abstract

Ribonucleotide reductases (RNRs) catalyze the reduction of ribonucleotides to the corresponding deoxyribonucleotides, used in DNA synthesis and repair. Two different mechanisms help deliver the required electrons to the RNR active site. Formate can be used as reductant directly in the active site, or glutaredoxins or thioredoxins reduce a C-terminal cysteine pair, which then delivers the electrons to the active site. Here, we characterized a novel cysteine-rich C-terminal domain (CRD), which is present in most class II RNRs found in microbes. The NrdJd-type RNR from the bacterium *Stackebrandtia nassauensis* was used as a model enzyme. We show that the CRD is involved in both higher oligomeric state formation and electron transfer to the active site. The CRD-dependent formation of high oligomers, such as tetramers and hexamers, was induced by addition of dATP or dGTP, but not of dTTP or dCTP. The electron transfer was mediated by an array of six cysteine residues at the very C-terminal end, which also coordinated a zinc atom. The electron transfer can also occur between subunits, depending on the enzyme's oligomeric state. An investigation of the native reductant of the system revealed no interaction with glutaredoxins or thioredoxins, indicating that this class II RNR uses a different electron source. Our results indicate that the CRD has a crucial role in catalytic turnover and a potentially new terminal reduction mechanism and suggest that the CRD is important for the activities of many class II RNRs.

## Introduction

Ribonucleotide reductases (RNRs)^4^ catalyze the reduction of ribonucleotides to the corresponding deoxyribonucleotides, which are required for DNA synthesis and repair. Because this reaction is the only known biological pathway for *de novo* deoxyribonucleotide synthesis, RNRs are considered to be essential for every living organism. Today, three different classes of RNRs are known, which all share a common fold and a general reaction mechanism based on a thiyl radical (1). All known RNRs, except a few viral RNRs, also share an allosteric substrate-specificity regulation, where binding of a dNTP or ATP molecule modulates the specificity of the active site of the enzyme (1). The three classes share a common ancestor (2); the main difference between them is their mechanism of radical generation (1, 3). With the different radical, generating mechanisms follow differences in dependence of oxygen and oligomeric composition.

Class I RNRs (NrdAB/NrdEF) generate the radical in a separate subunit (β, NrdB/F) via a dimetal center that is oxidized by molecular oxygen. The radical is then transferred to the catalytic subunit (α, NrdA/E) and back after the catalytic cycle. Thus, this class is oxygen-dependent and forms active α_2_β_2_ heterotetramers. Terminal reduction of the oxidized active site in α is performed by thioredoxin or glutaredoxin systems at the end of each turnover (4, 5).

Class III RNRs (NrdD) also require a separate subunit (NrdG) to generate the radical. An iron–sulfur cluster in NrdG confers homolytic cleavage of the cofactor *S*-adenosyl-l-methionine (AdoMet) that generates a stable glycyl radical in the catalytic subunit. This glycyl radical is extremely sensitive toward oxygen, leading to protein backbone cleavage at oxygen exposition. NrdDs are described to be active as α_2_ homodimers and NrdG is only needed to generate the glycyl radical in NrdD (6, 7). Formate was shown to be a terminal reducing agent for some NrdD enzymes (8). Recently, class III RNRs that use a thioredoxin system for terminal reduction have been described (9).

The RNR that is the focus of the current study, class II (NrdJ), generates the radical via homolytic cleavage of the vitamin B_12_ coenzyme (AdoCbl) within the enzyme, close to the active site. After the catalytic cycle, AdoCbl is reconstituted (10). This mechanism makes NrdJ enzymes independent of oxygen, but instead dependent on B_12_. The oligomeric organization of the NrdJ enzymes is more diverse than in the other classes. Some representatives, such as the enzyme from *Thermoplasma acidophilum*, have been shown to be active as α_2_ homodimers (11); the NrdJ from *Lactobacillus leichmannii* is active as monomer, where an additional domain mimics the dimer interface, required for allosteric regulation (12). Higher oligomers have been observed for the full-length NrdJ from *Thermotoga maritima*, whereas a truncated variant utilized for X-ray crystallography crystallized as a dimer (13). The terminal reductant is poorly studied for NrdJ enzymes. The monomeric NrdJ from *L. leichmannii* was shown to receive electrons from thioredoxin (14), but for other NrdJ enzymes reduction by thioredoxin or glutaredoxin has not been studied.

In the monomeric NrdJ, as well as in class I RNRs, the interaction with thioredoxin or glutaredoxin is mediated via a conserved C-terminal cysteine pair. Because of its position on a flexible C-terminal tail, this reduced cysteine pair is believed to have access to the active site and to re-reduce the disulfide that is oxidized during the nucleotide reduction reaction, in turn forming a disulfide that is subsequently reduced by the terminal reductant (4, 5). This study reports that most nonmonomeric class II RNRs contain a so far undescribed cysteine-rich C-terminal domain (CRD). We show that the CRD is involved in oligomerization and the terminal reduction of the class II RNR from *Stackebrandtia nassauensis* and that the CRD hence plays a crucial role in the catalytic cycle of class II RNRs.

## Results

### Phylogenetic analysis

**Figure 1.**
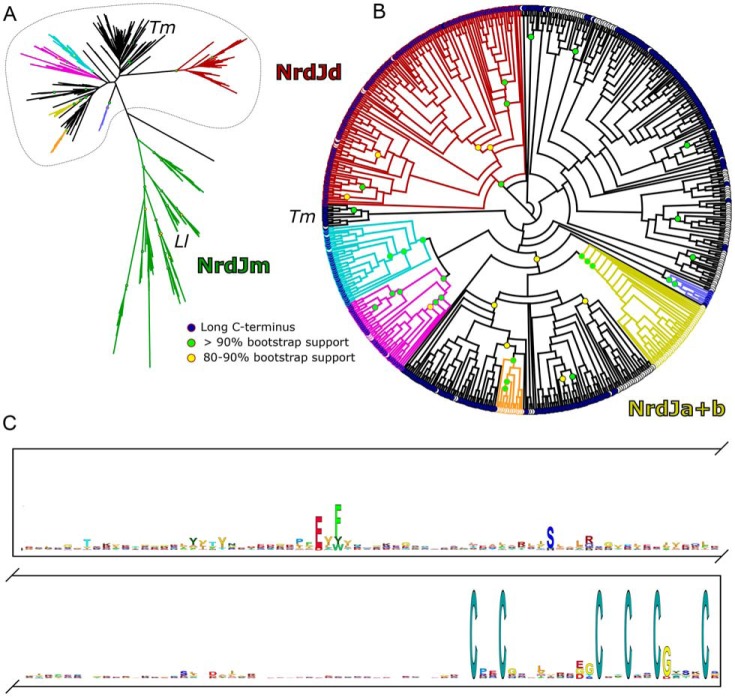
**Abundance and sequence of CRD were studied by phylogenetic and sequence analysis of NrdJ and NrdJd.** *A*, maximum likelihood phylogenetic tree of a representative selection of NrdJ sequences. Subclasses with characterized members (NrdJm (*green*), NrdJd (*red*), and NrdJa+b (*yellow*)) and candidate subclasses (*cyan*, *purple*, *orange*, and *light blue*) are indicated with color. *Lactobacillus leichmannii* RNR (*Ll*) is located in subclass NrdJm, whereas the RNR from *Thermotoga maritima* (*Tm*) is not part of a defined subclass. *B*, appearance of the cysteine-rich domain mapped on a phylogenetic tree of a selection of nonmonomeric NrdJ sequences. The selected subset is marked by the dotted line in *A*. Subclasses with characterized members (NrdJd (*red*) and NrdJa+b (*yellow*)) and candidate subclasses (*cyan*, *purple*, *orange*, and *light blue*) are indicated with color. The presence of a CRD is indicated with a *blue circle*, absence with a *white circle*. The NrdJa+b subclass has the encoded CRD as a separate gene. *C*, logo of the CRD derived from 217 nonredundant sequences from subclasses NrdJd and unclassified NrdJ.

We estimated a maximum likelihood phylogenetic tree from an alignment of sequences representing the full diversity of NrdJ sequences at the 75% identity level (Fig. 1*A*; supplemental data). The resolution increased after exclusion of sequences from the monomeric class II enzymes, subclass NrdJm, allowing us to define two well-supported, likely monophyletic subclasses (NrdJd, NrdJa+b) with at least one enzymatically characterized member (Fig. 1*B*; supplemental data; http://rnrdb.pfitmap.org).^5^ In addition, four likely monophyletic, well-supported, candidate subclasses and a number of unclassified sequences were identified. Subclass NrdJm contains the monomeric enzyme from *L. leichmannii* (12), subclass NrdJd contains the enzyme from *Streptomyces clavuligerus* (15), and subclass NrdJa+b contains the split enzyme from *Pseudomonas aeruginosa* (16, 17). Other well-characterized NrdJ enzymes such as the one from *T. maritima* and *T. acidophilum* are among the unclassified sequences (11).

An alignment of all NrdJ sequences shows major differences in the C-terminal region. A cysteine-rich C-terminal domain is present in 70% of the investigated sequences. The CRD is 150–220 amino acids long and contains hydrophobic regions with single conserved hydrophilic residues (Fig. 1*C*). The most striking feature of this domain is an array of six fully conserved cysteine residues at the C-terminal end of the sequence (Fig. 1*C*). No sequence similarity between the class I C-terminal sequence, which contains a single pair of conserved cysteines, and the NrdJ CRD was detected.

The CRD is predominant in subclass NrdJd and in three of the four candidate subclasses as well as in a majority of the unclassified sequences, but not at all in NrdJm. The NrdJa+b subclass consists of enzymes where the CRD is not genetically fused to the catalytic domain, but encoded by a separate gene, *nrdJb*. In some *Frankia* species and a few Alphaproteobacteria, two NrdJd enzymes are present where only one contains the CRD (supplemental Table S1; http://rnrdb.pfitmap.org).^5^

### Recombinant expression of candidate enzymes from subclass NrdJd

**Figure 2.**
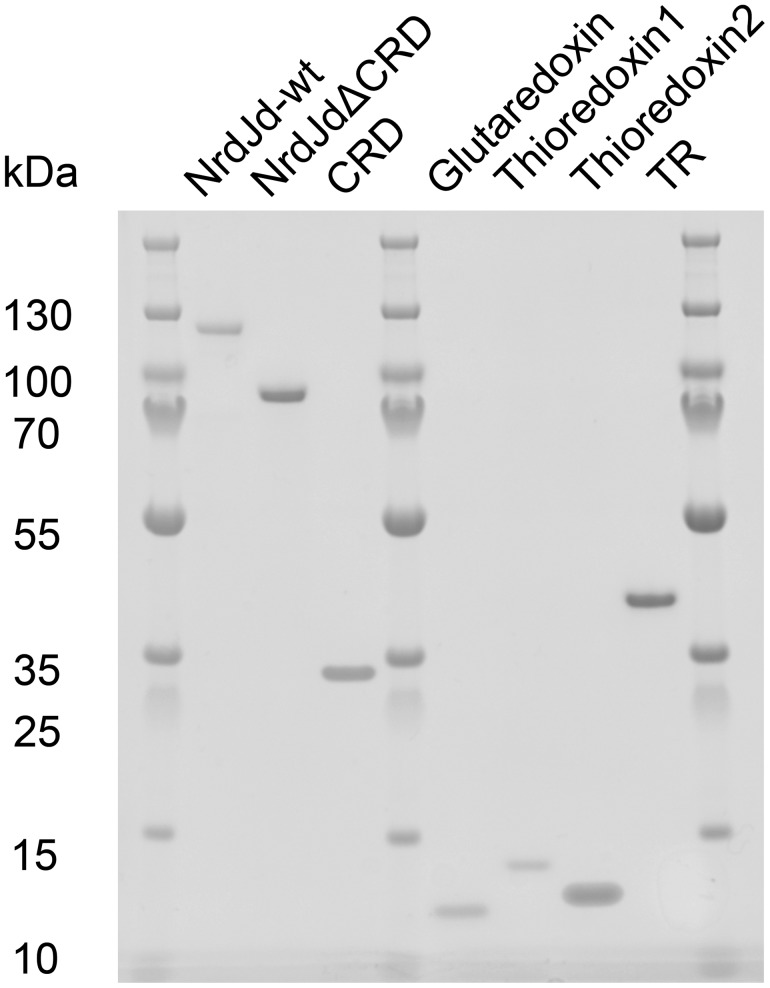
**Target enzymes from *S. nassauensis* are heterologously expressed and purified.** Electrophoresis was performed with a 4–12% Bis-Tris Protein Gel. NrdJd-wt (Snas 4560): full-length NrdJd enzyme (1–947); NrdJdΔCRD: C-terminal truncation without CRD (1–714); CRD: CRD of NrdJd (715–947); glutaredoxin (Snas 1785); thioredoxin 1 (Snas 2647); thioredoxin 2 (Snas 6430); TR: thioredoxin reductase (Snas 6431).

To study the properties and the function of the NrdJ subclass in general and the CRD in particular, a set of candidate proteins were identified using a bioinformatic prescreen to estimate successful expression of soluble protein. The NrdJd enzymes from *Streptomyces clavuligerus*, *Coraliomargarita akajimensis*, and *S. nassauensis* were selected and recombinantly expressed in *Escherichia coli*. Only the *S. nassauensis* NrdJd showed a sufficient amount of soluble expression of active enzyme with an intact CRD (Fig. 2).

In addition, both the isolated catalytic domain of the *S. nassauensis* enzyme (positions 1–714; NrdJdΔCRD), as well as the CRD alone (positions 715–947) were cloned and expressed in *E. coli* separately (Fig. 2). The expression of the CRD yielded negligible amounts of soluble protein and aggregation occurred quickly. From the full-length enzyme NrdJd-wt and the isolated catalytic domain NrdJΔCRD, soluble protein was obtained of high purity and in sufficient quantity. Initial activity assays showed that the enzyme was able to reduce CDP but not CMP or CTP.

### Allosteric regulation

**Figure 3.**
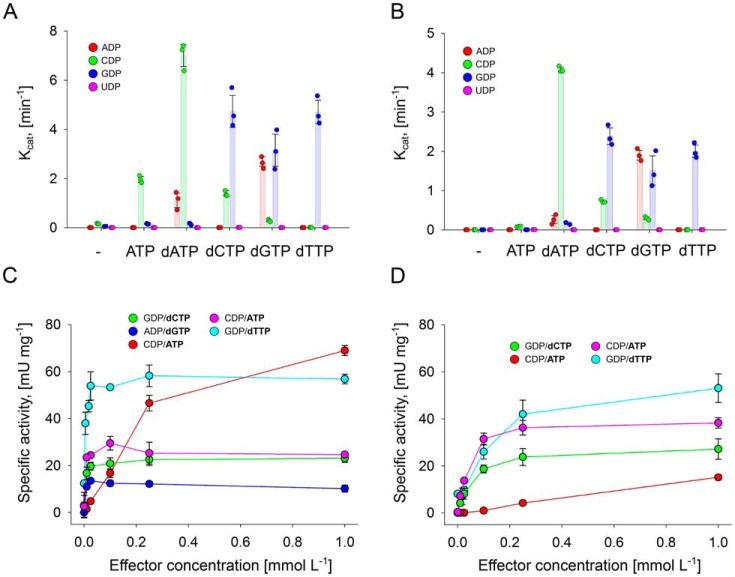
**The presence of effector molecules modifies the activity profiles of NrdJd-wt and NrdJdΔCRD.** *A* and *B*, four-substrate assay for NrdJd-wt (*A*) and NrdJdΔCRD (*B*) with or without effector. Each reaction contains the four potential substrates ADP, CDP, GDP, and UDP plus one of the potential effectors dATP, dCTP, dGTP, dTTP, or ATP. *C* and *D*, influence of the effector concentration on enzyme activity of NrdJd-wt (*C*) and NrdJdΔCRD (*D*) with one of the potential effectors dATP, dCTP, dGTP, dTTP, or ATP. Titrations were performed in a concentration range from 4 to 1000 μmol liter^−1^, including measurements without effector. Data were obtained in three independent experiments. *Error bars* indicate the mean ± S.D.

An important characteristic of all RNRs known so far, with the exception of class I RNRs from herpes viruses (18), is the allosteric regulation of substrate specificity. Thereby, effector binding in the specificity site in the dimer interface influences the substrate specificity of the active site. The potential effectors ATP, dATP, dCTP, dGTP, and dTTP were tested for their influence on the reduction of ADP, CDP, GDP, and UDP by NrdJd-wt and NrdJdΔCRD. All four potential substrates were present in the assay together with one of the five potential effector molecules at a time, and conversion of all substrates was measured. The effectors ATP and dATP activated the wild-type enzyme for CDP reduction (Fig. 3*A*). dCTP and dTTP enhance the activity for GDP reduction, whereas dGTP activates both enzymes for ADP and GDP reduction. UDP conversion was not observed with any of the tested effector nucleotides. Except for a low activity with ATP as effector, the experiment yielded similar results for NrdJdΔCRD (Fig. 3*B*).

To investigate the affinity of NrdJd-wt and NrdJdΔCRD toward the preferred effectors, activity assays with a single substrate were performed in the presence of increasing effector concentrations. For NrdJd-wt, all effectors yielded nearly maximal activity already at the lowest tested effector concentration of 4 μmol liter^−1^ (Fig. 3*C*). This concentration represents only a 2-fold molar excess over the applied enzyme concentration of 2 μmol liter^−1^. This indicates very low K_L_ values (<4 μmol liter^−1^), but it also means that the actual values cannot be determined based on activity at these conditions. Thereby, the *K*_L_ value is defined as the effector or ligand concentration where half of the maximal reaction velocity is achieved. For NrdJdΔCRD the effector titrations yielded saturation curves with K_L_ values between 50 and 150 μmol liter^−1^ (Fig. 3*D*). In conclusion, the affinity of the C-terminal truncated enzyme toward each tested effector is reduced. However, the overall pattern of activation or inactivation of the enzyme for different substrates by the effectors is intact.

### Oligomeric state

**Figure 4.**
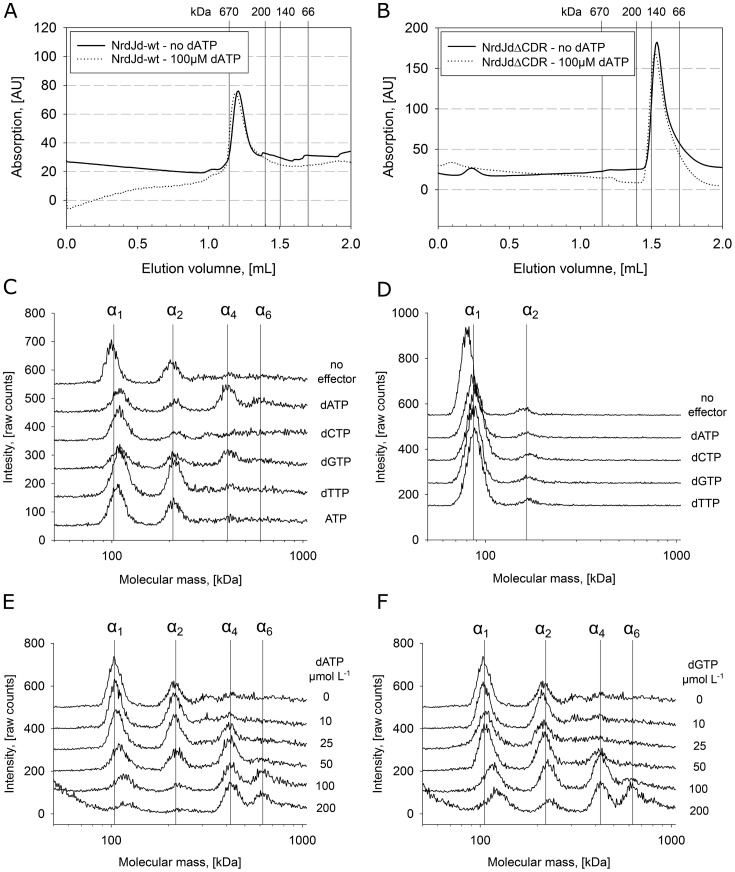
**Effector molecules influence the oligomeric state of NrdJd.** *A* and *B*, size exclusion chromatography of NrdJd-wt (*A*) and NrdJdΔCRD (*B*) with 1 mg ml^−1^ protein each. *C*, GEMMA experiments with 0.075 mg ml^−1^ NrdJd-wt and 100 μmol liter^−1^ of one of the potential effectors. *D*, GEMMA experiment with 0.025 mg ml^−1^ NrdJdΔCRD and 100 μmol liter^−1^ of one of the potential effectors. *E*, GEMMA experiment with 0.075 mg ml^−1^ NrdJd-wt and a titration of the dATP concentration. *F*, GEMMA experiment with 0.075 mg ml^−1^ NrdJd-wt and a titration of the dGTP concentration. All GEMMA experiments represent five separate measurements for each condition.

The oligomeric state of *S. nassauensis* NrdJd was studied using size exclusion chromatography (SEC) at high protein concentration (1.0 mg ml^−1^) and gas phase electrophoretic macromolecule analysis (GEMMA) at a lower concentration (0.075 mg ml^−1^). The latter is comparable to the enzyme concentration in the activity assays. The full-length NrdJd yielded a SEC peak at 450 kDa, corresponding to a tetramer that was unaffected by dATP addition (Fig. 4*A*). In GEMMA, NrdJd-wt shows a mixture of monomers and dimers that shifts to a tetramer only in the presence of 100 μmol liter^−1^ dATP (Fig. 4*C*). NrdJdΔCRD, on the other hand, is present as a monomer between 90 and 100 kDa both in SEC and in GEMMA (Fig. 4, *B* and *D*). The NrdJdΔCRD monomer and the small fraction of dimer visible in GEMMA were not affected by the addition of dATP (Fig. 4*D*). dGTP yielded results similar to dATP, whereas addition of dTTP (Fig. 4*C*) and dCTP induced dimerization but not tetramerization in NrdJd-wt. In titrations with dATP, as well as dGTP, NrdJd-wt was in a monomer–dimer equilibrium at lower concentrations of the effector and gradually changed to tetramers and even hexamers when the effector concentration was increased (Fig. 4, *E* and *F*). By interpolation of the quantified GEMMA results based on mass concentration, half the enzyme is present as tetramer or hexamer at a concentration of 17 μmol liter^−1^ dATP.

### Electron transfer

**Figure 5.**
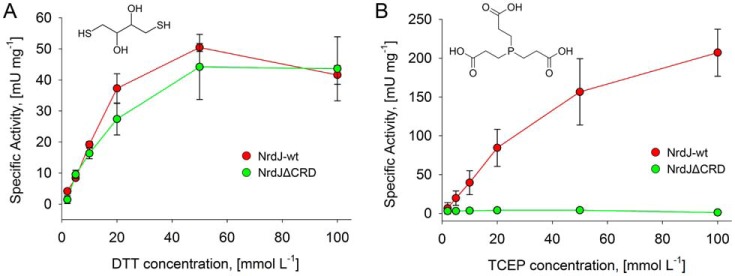
**RNR activity of NrdJd-wt and NrdJdΔCRD depends on the applied reductant.** *A* and *B*, enzyme activity of NrdJd-wt (*red*) and NrdJdΔCRD (*green*) in the presence of different concentrations of the reductants DTT (*A*) and TCEP (*B*). Assays were performed with CDP as substrate and dATP as effector. All experiments were performed in triplicate. *Error bars* indicate the mean ± S.D.

**Figure 6.**
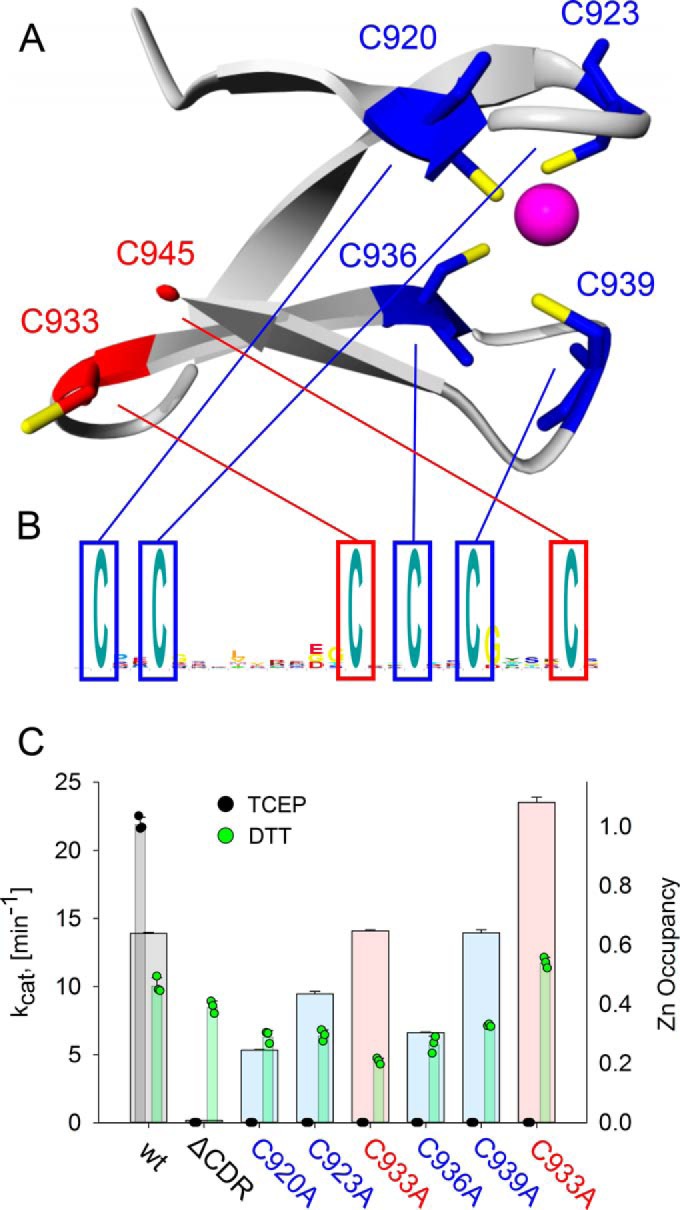
**A C-terminal zinc–binding site is elucidated by homology modeling and biochemically characterized.** *A*, homology model of the last 30 amino acids of the CRD from *S. nassauensis* NrdJd, based on the crystal structure of tRNA(Ile2) 2-agmatinylcytidine synthetase (PDB ID: 4RVZ) (20). *B*, logo of the C-terminal end of *S. nassauensis* NrdJd-wt. *C*, activities with the reductant TCEP (*narrow bars*; *black*) and DTT (*narrow bars*; *green*) and zinc content (*broad bars*) of NrdJd-wt and the cysteine to alanine variants. The colors *red* and *blue* refer to the predicted location of the respective cysteine residue. Data were obtained in three independent experiments. *Error bars* indicate the mean ± S.D.

Because two C-terminal cysteine residues are known to be the acceptors of electrons from glutaredoxins and thioredoxins in the class I and NrdJm enzymes, the role in electron transfer of the *S. nassauensis* NrdJd CRD and its six cysteine residues was investigated. Earlier studies with alternative reducing systems have demonstrated that the phosphine TCEP specifically reduces the C-terminal cysteine residues of class I RNR, whereas DTT can reduce the enzyme active site directly (19). In this study, *S. nassauensis* NrdJd-wt and NrdJdΔCRD were tested with both reductants (Fig. 5). NrdJd-wt activity is supported by both DTT and TCEP, with 4-fold higher specific activity using the latter. NrdJdΔCRD exhibits a comparable activity with the reductant DTT as the wild-type enzyme, but is completely inactive with TCEP. An attempted reconstitution of the full-length protein with equimolar amounts of NrdJdΔCRD and the separately expressed CRD did not show any activity with TCEP (data not shown). A PROSITE search did not fit the *S. nassauensis* CRD to any known cysteine-rich motif such as zinc fingers. On the other hand, a sequence similarity search against the Protein Data Bank database suggested similarity to a zinc-binding site from tRNA(Ile2) 2-agmatinylcytidine synthetase (PDB ID: 4RVZ) from *Archaeoglobus fulgidus* (20). Based on this structure, a homology model of the *S. nassauensis* C-terminal 35 residues was created applying SWISS-MODEL (Fig. 6*A*).

The model suggested that a zinc is coordinated by the cysteine residues Cys-920, Cys-923, Cys-936, and Cys-939 (Fig. 6*B*). The remaining two cysteine residues Cys-933 and Cys-945 are in close proximity in the model and could form the site of terminal reduction. This homology model was applied as a starting model for subsequent experimental confirmation. Accordingly, six different cysteine to alanine substitution variants of *S. nassauensis* NrdJd were constructed and their enzyme activity tested in assays applying either DTT or TCEP as reductants. All variants retained at least 50% of their specific activity with the reductant DTT (Fig. 6*C*). However, none of the variants besides the wild type showed any enzyme activity with the reductant TCEP, suggesting all cysteines are essential for active site reduction.

In an attempt to assess the four cysteine residues that are suggested to coordinate zinc, inductively coupled plasma (ICP) measurements were performed for the wild-type enzyme and all six cysteine to alanine mutants (Fig. 6*C*). The wild-type enzyme as well as the variants C933A and C939A showed a zinc occupation of more than 60% per monomer. The variants C920A, C923A, and C936A yielded reduced occupancy to between 20 and 40%, whereas C945A was fully occupied. Thus, the exchange of the two cysteine residues predicted to form the terminal cysteine pair had comparable or higher Zn^2+^ occupation than the wild type, whereas of the four cysteine residues predicted to coordinate the zinc, three showed a reduced zinc content, and one was equal to the wild type.

### Subunit cross talk

**Figure 7.**
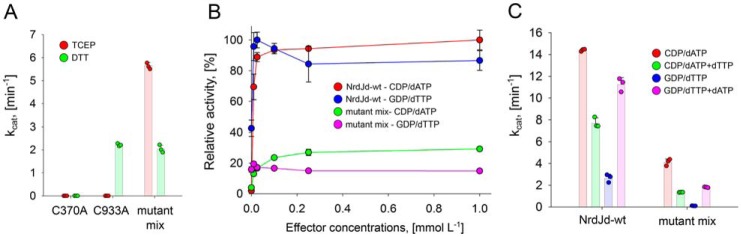
**Electron transfer between subunits is observed in mutant mixtures of NrdJd.** *A*, specific activity per monomer of the C933A and C370A proteins tested individually and in an equimolar C933A/C370A mixture (mutant mix). *B*, relative activities of NrdJd-wt and the mutant mix in effector titrations with the substrate effector pairs CDP/dATP (100% equals *k*_cat_ = 15.3 min^−1^) and GDP/dTTP (100% equals *k*_cat_ = 3.1 min^−1^) and TCEP as reductant. *C*, activities of NrdJd-wt and the mutant mix with the substrates CDP or GDP and effector mixtures. TCEP was applied as reductant. Concentrations: dATP 250 μmol liter^−1^, dTTP 1 mmol liter^−1^. Data were obtained in three independent experiments. *Error bars* indicate the mean ± S.D.

The CRD is responsible for electron delivery to the active site but as shown above, the NrdJd can adopt different oligomeric states. This leads to the question whether the C-terminal cysteine residues can only reach the active site of their own subunit or if they can also deliver electrons to other subunits within the oligomer. To investigate this, an active site cysteine-deficient mutant (C370A) was mixed in equimolar amounts with a C-terminal mutant protein. In control experiments, the C370A mutant showed no activity with any of the reductants, TCEP or DTT, whereas the C-terminal mutant C933A was active with DTT but not with TCEP (Fig. 7*A*). Yet the mixture of the two mutant proteins exhibited high enzyme activity with both DTT or TCEP, indicating electron transfer from the intact C terminus of the active site mutant C370A to the active site of the CRD mutant C933A.

Because of the complex oligomeric organization of NrdJd-wt, the influence of the oligomeric state on the subunit cross talk was investigated. Activity assays were performed with the wild-type enzyme and the mixture of the CRD mutant C933A and active site mutant C370A. To test for cross talk in the dimer, the substrate/effector pair GDP/dTTP was applied, where no formation of higher oligomers was observed in the previous experiments. Cross talk in higher oligomers was investigated using the substrate/effector pair CDP/dATP, where formation of tetramers and hexamers was shown in the GEMMA experiments (Fig. 7*B*). In both cases TCEP was applied as reductant. NrdJd-wt had a low K_L_ value <4.0 μmol liter^−1^ for dATP. For the C933A/C370A mixture, an increased K_L_ value of 15.0 ± 4.5 μmol liter^−1^ was determined. The *V*_max_ value in the variant mixture was reduced to 30% of the wild type, which is close to the theoretical maximum value of 50% considering that half of the subunits in the mixture have a defective active site. For the effector dTTP, NrdJd-wt showed maximum activity already at the lowest dTTP concentration, indicating a high affinity. The C933A/C370A mixture exhibited very low overall activity between 3 and 7% of NrdJd-wt with no significant effect of increasing dTTP concentrations. To further assess the effect of dATP, assays were performed with mixtures of dATP and dTTP (Fig. 7*C*). For both the C933A/C370A mixture and NrdJd-wt, activity with the substrate CDP was lower in the presence of dATP plus dTTP compared with dATP alone. However, the addition of 250 μmol liter^−1^ dATP to 1 mmol liter^−1^ dTTP led to an increase in GDP reduction for the C933A/C370A mixture and NrdJd-wt. The effect was especially pronounced in the C933A/C370A mixture, where activity increased 18-fold compared with 4-fold in the wild-type enzyme.

### Native electron donor

**Figure 8.**
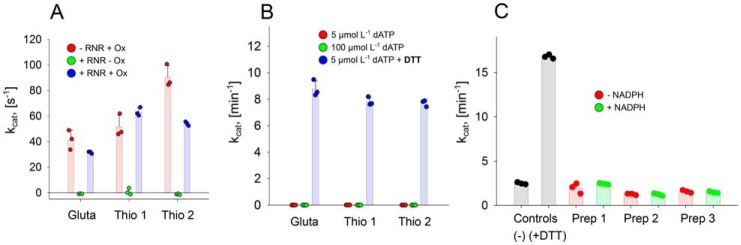
**No electron transfer from potential native electron donor systems can be observed in activity measurements.** *A*, specific activity of glutaredoxin and the thioredoxins in the presence or absence of NrdJd-wt (+RNR and −RNR), respectively. The assays were performed with or without additional oxidant used as substrate (+Ox or −Ox). The additional oxidants were insulin for the thioredoxins and HED for glutaredoxin. *B*, specific activity of NrdJd-wt in combination with glutaredoxin and the thioredoxins with the substrate CDP. *C*, activity assays with NrdJd-wt in the presence of cell lysate preparations from *S. nassauensis* with (*green*) or without (*red*) 250 μmol liter^−1^ NADPH addition: protein-free lysate (*Prep 1*), cleared lysate (*Prep 2*), and complete lysate (*Prep 3*). No artificial reductant such as DTT was added. As positive and negative controls, RNR-assays with (+*DTT*) and without (−) the reductant DTT were performed. RNR activity in the preparations and controls was determined by HPLC measurement of CDP consumption and dCDP synthesis. All data were obtained in three independent experiments. *Error bars* indicate the mean ± S.D.

To further investigate the role of the C-terminal cysteine residues as terminal reducing sites of the enzyme, we attempted to define the native reductant. In many class I RNRs as well as for the NrdJm from *L. leichmannii*, thioredoxins and glutaredoxins serve as native reductants (14, 1). Therefore, we performed a sequence similarity search for thioredoxins and glutaredoxins in the *S. nassauensis* proteome. The organism encodes two thioredoxins, Snas 2647 (thioredoxin 1) and Snas 6430 (thioredoxin 2), as well as one glutaredoxin, Snas 1785. The three redoxins, together with the thioredoxin reductase (TR) Snas 6431, were cloned and recombinantly expressed in *E. coli*. All constructs yielded soluble protein in high amounts with high specific activities on artificial substrates (Fig. 8*A*). All thioredoxins and glutaredoxins were tested in the RNR activity assay together with their corresponding electron source (thioredoxin reductase + NADPH for the thioredoxins, and glutathione + glutathione reductase + NADPH for glutaredoxin). However, no RNR activity was detected in presence of any of the redoxin systems (Fig. 8*B*). To test whether the conditions for the combined assay are suitable for RNR and the redoxin systems, both reaction systems were monitored separately. Addition of the artificial reductant DTT to the full reaction mix resulted in high specific activity of the RNR (Fig. 8*B*). Addition of the artificial electron acceptors insulin (thioredoxins) and 2-hydroxyethyl disulfide (HED) (glutaredoxin) yielded high specific activities for the redoxin systems (Fig. 8*A*). Both the RNR and the redoxin systems exhibited high activity at the combined assay conditions, but no transfer of electrons could be observed between them.

In another attempt to identify the native electron donor, RNR assays were performed with different types of *S. nassauensis* cell lysates (Fig. 8*C*). Tests were performed with 1) protein-free lysate, 2) cleared lysate, and 3) complete lysate. None of the lysates increased activity beyond the level measured in the negative control.

## Discussion

We characterized a unique C-terminal cysteine-rich domain, present in a majority of class II RNRs, which is involved in oligomerization and terminal reduction during catalysis. This domain is identical to the NrdJb subunit in the NrdJa+b split enzyme described for *P. aeruginosa* (16, 17). and the CRD that was spontaneously cleaved from *T. maritima* NrdJ, used for crystallization (13). Sequence analysis revealed the presence of the CRD in 70% of the nonredundant NrdJ sequences, excluding the monomeric NrdJm which never contains the CRD (Fig. 1*B*; supplemental Table S1). Among the nonmonomeric NrdJs, the presence of the CRD does not follow a simple phylogenetic pattern (Fig. 1, *A* and *B*). The domain is present in the majority of subclasses and candidate subclasses. Absences are spread throughout the tree, suggesting independent losses of the domain. In NrdJd, the only absences of the CRD we could detect were from genomes also encoding another NrdJd with a CRD.

The high occurrence of the CRD domain in NrdJ enzymes, as well as its genetic separation from the catalytic subunit in NrdJa+b, indicates not only the general importance of the domain but also that it is a separately folded independent domain. Previous studies of enzymes containing the CRD have suffered from the aggregation-promoting properties of this domain as well as its susceptibility to cleavage (11, 17). For the genetically separate CRD, NrdJb from *P. aeruginosa*, it was not possible to obtain soluble protein by heterologous expression in *E. coli* (17). Recombinant expression of the full-length unclassified NrdJ from *T. maritima* yielded soluble protein but the CRD was partially cleaved during expression (13). Fortunately, the NrdJd from *S. nassauensis* did not show any sign of cleavage during expression or further experiments.

Our functional analysis of the CRD suggests an important role in oligomerization and electron transfer. The CRD-dependent formation of higher oligomers is well in line with observations made for the NrdJ from *T. maritima*, which was first characterized as a higher oligomer with about 450 kDa (11). The C-terminally truncated version was later characterized as a dimer (13). In this study, we show that the C-terminally truncated NrdJ enzyme from *S. nassauensis* is in a monomer–dimer equilibrium. Tetramers and hexamers were only observed in the full-length enzyme, showing the importance of the CRD for the formation of higher oligomers. Experiments also showed that this oligomerization is stimulated by addition of the purine nucleotides dATP and dGTP, but not by the pyrimidine nucleotides dTTP and dCTP.

Although the actual mechanism behind the CRD's role in oligomerization and how this affects activity of the enzyme still remains to be described, the role of the CRD in the re-reduction of the active site cysteine residues is much clearer. Although sequence similarity between the CRD of most NrdJs on one side and the C termini of class I RNRs and monomeric NrdJs on the other side is limited to a single pair of cysteines, the mechanism appears comparable (4, 5, 14). This is supported by the inability of NrdJdΔCRD to accept TCEP as terminal reductant, whereas activity is retained with DTT, the latter being small enough to directly reduce the active site and the former is not (19).

The TCEP experiments with the six cysteine to alanine exchanges showed that all six cysteine residues are necessary for the functionality of the electron transfer. Homology modeling indicated that four of these cysteine residues (Cys-920, Cys-923, Cys-936, Cys-939) could be involved in zinc binding whereas the remaining two (Cys-933, Cys-945) appear to form the redox-active cysteine pair. However, the ICP measurement only shows reduced zinc content in three of the four predicted positions. A zinc occupancy between 0.2 and 0.4 in the variants, compared with 0.6 in the wild-type enzyme, is in good agreement with values measured in comparable studies (21, 22). It is noteworthy that both C933A and C939A show the same zinc content as the wild type, whereas it is reduced in C936A. Possibly, the zinc can be coordinated either by Cys-933 and Cys-936 or Cys-936 and Cys-939. Both these pairs have the common sequence C-*X-X*-C that is typical of zinc ligating cysteines. A certain promiscuity in the coordination of the zinc would also explain why no single mutant showed a complete loss of zinc as observed in the full truncation. Taking the potential swap between Cys-933 and Cys-939 into account, the results are consistent with the homology model. We thus propose that the residues Cys-920, Cys-923, Cys-936, and Cys-939 coordinate a zinc residue, structuring the C-terminal end and bringing Cys-933 and Cys-945 in close proximity to each other. This pair can then act as an electron shuttle between the electron donor and the active site. Coordination of the zinc by Cys-933 in case of the C939A variant seems possible, in which case a restructured metal-binding site could still bind the zinc ligand, but Cys-945 would be deprived of its partner, prohibiting the role of terminal reduction.

A C-terminal zinc-binding site was previously described for NrdD enzymes (23, 24). It was proposed to be a structure forming element, which keeps the catalytically crucial glycyl radical in correct orientation. Because of the low sequence similarity and the very different location of the zinc-binding site within the sequences, there is no evidence for homology between the sites in class II and class III RNRs. Cysteine-coordinated zincs with a structural role are commonly found in proteins (25, 26) and are often the result of convergent evolution.

With the help of mutant proteins defective in the active site (C370A) and in the CRD (C933A), we demonstrated electron transfer from the CRD of one subunit to the active site of another subunit. The effects of oligomerization on this cross talk were investigated by addition of the effectors dATP and dTTP with their different oligomerization effects. The results indicate that an efficient transfer of electrons between the subunits can occur only in tetramers or higher oligomers. The measured K_L_ value for dATP of 15 μmol liter^−1^ compares well with the GEMMA results where, at an estimated concentration of 17 μmol liter^−1^, half of the enzyme is present as tetramer or higher oligomer. Cross talk between terminal reduction sites has been observed before in the class I RNR from *S. cerevisiae* and suggested to occur within the dimer (27). Higher oligomerization that allows for tuning of electron transfer between the subunits could represent a novel layer of activity regulation.

In GEMMA experiments and activity assays all four dNTPs were shown to induce dimerization and act as effectors via the specificity site but the formation of higher oligomers was induced only by dATP and dGTP. We therefore sought to study whether there is an additional effector-binding site, different from the specificity site. Attempted direct binding assays and isothermal titration calorimetry (ITC) experiments did not yield conclusive results (data not shown), but experiments with mixtures of effectors were indicative of a third nucleotide-binding site. Although addition of dTTP to a CDP/dATP assay inhibited enzyme activity in accordance with the described allosteric regulation, addition of dATP to a GDP/dTTP assay instead led to an increase in activity. However, dATP does not activate GDP reduction via the described allosteric mechanism. Instead, it suggests that dATP binding to another site may mediate the increase in activity by formation of higher oligomers.

To assess the physiological role of the CRD, we attempted to find the native electron donor of the system. The three tested glutaredoxins and thioredoxins from *S. nassauensis* were not able to supply the RNR with electrons under the tested conditions. The observation that all systems separately, the RNR and the glutaredoxin-thioredoxin systems, are active but cannot exchange electrons, questions whether RNRs with the CRD are dependent on reduction by a redoxin system at all. Attempts to find the native electron donor in *S. nassauensis* cell lysates did not, however, yield any positive results. Because the organism also contains a class I RNR, the native electron donor system might not be expressed during the employed culture conditions or might be inactivated in the lysis process. As shown by Berardi and Bushweller (28) in NMR studies, the very flexible, disordered C-terminal tail of class I and NrdJm enzymes plays a vital role in the interaction of the C-terminal cysteine residues with the redoxins. The highly ordered C terminus in CRD-containing NrdJ enzymes might be unsuitable for an interaction with redoxins in a fashion comparable to class I or NrdJm RNRs. Our so far unproductive attempts to find the reducing system for *S. nassauensis* together with the recent discovery that some class III RNRs are indeed reduced by redoxins (9), and not by formate that was long believed to be the sole reductant of these enzymes, show the plurality of ultimate reductants that can be involved in ribonucleotide reduction.

## Experimental procedures

### Bioinformatics

All full-length NrdJ sequences in RefSeq were downloaded from the Ribonucleotide Reductase database (http://rnrdb.pfitmap.org)^5^ and clustered at 75% identity with USEARCH (29) to create a selection of representative sequences. Sequences were aligned with ProbCons (30), and reliable alignment positions were selected with BMGE (31) using the BLOSUM30 model. A phylogeny was estimated with RAxML (32) in rapid bootstrapping mode with automatic bootstopping (extended majority rule) using the PROTGAMMAAUTO model. A Hidden Markov Model profile for the CRD domain was constructed from the CRD domain found in 217 sequences in the alignment used for phylogenetic estimation. The Skylign server was used to graphically represent the profile as a sequence logo (33).

Secondary structure prediction was performed with the Chou & Fasman Secondary Structure Prediction Server (CFSSP) (34, 35). The homology model of the C terminus was constructed via the SWISS MODEL server (36–39). After automated template search, a model was built based on the template with the highest sequence identity. For the model discussed above, the 35 amino acid C termini of tRNA(Ile2) 2-agmatinylcytidine synthetase from *A. fulgidus* (PDB ID: 4RVZ) with a sequence identity of 31% was applied as template.

### Molecular cloning

The genes from the NrdJd from *S. nassauensis* (Snas 4560; full length and CRD) and *S. clavuligerus* (AJ224870) as well as the genes from the thioredoxins (Snas 6430, Snas 2647), the glutaredoxin (Snas 1785), and the thioredoxin reductase (Snas 6431) were amplified from genomic DNA via PCR. All genes were cloned into a pET22b(+) vector via NdeI and HindIII restriction sites adding an N-terminal His-tag connected via a thrombin restriction site. For recombinant expression, the vectors were transformed in *E. coli* BL21(DE3).

### Mutagenesis

The C-terminal truncation of the NrdJd from *S. nassauensis*, NrdJdΔCRD, as well as all cysteine to alanine mutations were performed via site-directed mutagenesis using mismatched oligonucleotides. For NrdJdΔCRD, the GAG bases coding for Glu-715 were exchanged by a TAA stop codon. For the cysteine to alanine mutations the TGC and TGA codons for cysteine were replaced by GCA coding for alanine.

### Recombinant expression and purification

Expression was performed in LB medium in 600-ml scale in baffled shake flasks. The medium was inoculated from a preculture to a starting *A*_600_ of 0.05–0.1 and incubated at 37 °C and 150 rpm. After reaching an *A*_600_ of 0.8, the temperature was decreased to 15 °C (NrdJds and truncations) or 20 °C (glutaredoxin, thioredoxins, and thioredoxin reductase). After induction with IPTG with a final concentration of 0.1 mmol liter^−1^, the cultures were incubated for 4 h (NrdJds and truncations) or 18 h (glutaredoxin, thioredoxins, and thioredoxin reductase). Cells were then harvested by centrifugation.

Cell lysis was performed in HisWash buffer (50 mmol liter^−1^ Tris, pH 7.5, 300 mmol liter^−1^ NaCl, 20 mmol liter^−1^ imidazole, 1 mmol liter^−1^ DTT) by high-pressure homogenization three times at 15 megapascals (MPa). Purification was performed via immobilized metal ion affinity chromatography (IMAC). The supernatant from the cell lysis was applied on a HisTrap column washed with HisWash buffer and eluted with HisElute buffer (50 mmol liter^−1^ Tris, pH 7.5, 300 mmol liter^−1^ NaCl, 500 mmol liter^−1^ imidazole, 1 mmol liter^−1^ DTT).

In a second purification step, elution fractions from the IMAC were loaded on HiLoad^®^ 16/600 Superdex^®^ 200 pg gel filtration column and run with SEC buffer (50 mmol liter^−1^ Tris, pH 7.5, 300 mmol liter^−1^ NaCl, 1 mmol liter^−1^ DTT). If not used immediately, purified proteins were frozen in liquid nitrogen and stored at −80 °C.

### Analytical size exclusion chromatography

SEC for analytical purposes was run on a Superdex^®^ 200 Increase 3.2 column. 25 μl of protein sample with a concentration of 1.0 mg ml^−1^ were injected into the column and run with SEC buffer (50 mmol liter^−1^ Tris, pH 7.5, 300 mmol liter^−1^ NaCl, 1 mmol liter^−1^ DTT) in the presence or absence of 100 μmol liter^−1^ effector.

### Gas phase electrophoretic mobility molecule analysis

GEMMA experiments were performed on TSI Electrospray Aerosol Generator 3480, TSI Electrostatic Classifier 3080, and TSI Ultrafine Condensation Particle Counter 3025A. The buffer of the protein samples was changed to 40 mmol liter^−1^ ammonium acetate (pH 7.5) via a desalting column. Substrates and effectors were added to the protein sample with an equimolar amount of MgCl_2_. The samples were run in the final conditions: 40 mmol liter^−1^ ammonium acetate (pH 7.8), 0.005% Tween 20, 0.2 mmol liter^−1^ DTT, 100 μmol liter^−1^ effector, 100 μmol liter^−1^ substrate. The final concentrations of the proteins applied were 0.075 mg ml^−1^ for *S. nassauensis* NrdJd full length and 0.025 mg ml^−1^ for NrdJdΔCRD. The samples were run at a pressure of 1.7 p.s.i., a current of 160–200 nanoamperes (nA), and a voltage of 1.8–2.0 kV. For data collection, five runs per sample were conducted.

### Enzyme activity assay

Specific enzyme activities were determined by measurement of the conversion of NDP to dNDP, catalyzed by the enzyme in the presence of different dNTPs as allosteric effectors. All enzymatic assays were performed in triplicates. Under standard conditions the assay contains 50 mmol liter^−1^ Tris (pH 8.0), 20 mmol liter^−1^ MgCl_2_, 50 mmol liter^−1^ DTT, 1.0 mmol liter^−1^ dATP, 2.0 mmol liter^−1^ CDP, 5.0 μmol liter^−1^ AdoCbl, and between 2 and 5 μmol liter^−1^ enzyme (0.2–0.5 mg ml^−1^). In effector titration experiments, a concentration range from 4 to 1000 μmol liter^−1^ was applied, including a measurement without effector. In the four substrate assays, 0.5 mmol liter^−1^ of each substrate (ADP, CDP, GDP, UDP) were applied with 0.5 mmol liter^−1^ of the tested effector. The assays, performed in 50-μl scale, were incubated 15 or 30 min at room temperature and stopped by addition of 50 μl methanol. After addition of 200 μl water, the samples were analyzed via HPLC. 20 μl of the sample were injected onto a C18 column and run with the following buffer: 50 mmol liter^−1^ potassium P_i_ (pH 7.5), 10% (v/v) methanol, 0.25% (v/v) tetrabutylammonium hydroxide. The buffer was prepared with KH_2_PO_4_ by setting the pH value with KOH. The analytes were eluted in a linear gradient of methanol from 10 to 30% (v/v).

Photometric assays for thioredoxins and glutaredoxins based on the artificial electron acceptors Insulin and HED were performed as described in earlier studies (40, 41). The thioredoxin assay contains 50 mmol liter^−1^ Tris (pH 8.0), 20 μmol liter^−1^ thioredoxin, 1 μmol liter^−1^ thioredoxin reductase, 750 μg ml^−1^ insulin, and 250 μmol liter^−1^ NADPH. The glutaredoxin assay contains 50 mmol liter^−1^ Tris (pH 8.0), 20 μmol liter^−1^ glutaredoxin, 7.5 units ml^−1^ glutathione reductase, 1 mmol liter^−1^ glutathione, 750 μmol liter^−1^ HED, and 250 μmol liter^−1^ NADPH. All assays were run in triplicate for 5 min at room temperature.

In the combined redoxin/RNR assays all reaction components for the single RNR and redoxin assays were combined with the following adjustments: 5 μmol liter^−1^ RNR, 5 mmol liter^−1^ glutathione, 20 μmol liter^−1^ thioredoxin/glutaredoxin. The assays were incubated for 30 min at room temperature and analyzed photometrically as well as by HPLC as described above.

### Inductively coupled plasma mass spectrometry

ICP measurements were performed at the Z.B.M.—Analytical Laboratory at the Hebrew University of Jerusalem. ICP-OES analysis was performed after an acidic hot block digestion. ICP-OES measurements were performed in triplicate applying yttrium as internal standard.

## Author contributions

The concept of the manuscript and the writing were performed by C. L., D. L., and B.-M. S. C. L. performed a large part of the experimental work. D. L. and B. M. S. performed the phylogenetic investigation of class II RNRs. V. R. J. and A. H. helped with the performance and interpretation of the GEMMA experiments. I. R. G. and M. S. contributed in the production and characterization of the studied enzymes. M. C. established and performed HPLC analytics and helped interpreting the results. All authors critically reviewed the manuscript and approved its publication.

## Supplementary Material

Supplemental Data

## Footnotes

5 Please note that the JBC is not responsible for the long-term archiving and maintenance of this site or any other third party hosted site.

## Footnotes

4 The abbreviations used are:

RNR: ribonucleotide reductase
AdoCbl: adenosylcobalamin
AdoMet: *S*-adenosyl-l-methionine
CRD: cysteine-rich C-terminal domain
GEMMA: gas phase electrophoretic macromolecule analysis
HED: 2-hydroxyethyl disulfide
ICP: inductively coupled plasma analysis
ICP-OES: inductively coupled plasma optical emission spectrometry
NrdJd-wt: the full-length version of the *Stackebrandtia nassauensis* class II RNR
NrdJdΔCRD: the C-terminally deleted version of the *S. nassauensis* class II RNR
SEC: size exclusion chromatography
TCEP: tris(2-carboxyethyl)phosphine hydrochloride
IPTG: isopropyl 1-thio-β-d-galactopyranoside.
