# Supplementary material for: A unique cysteine-rich zinc finger domain present in a majority of class II ribonucleotide reductases mediates catalytic turnover
Source: J Biol Chem. 2017 Oct 2;292(46):19044–54. doi: 10.1074/jbc.M117.806331 (PMC5704485; doi:10.1074/jbc.M117.806331)
Supplement: Supplemental Data [file supp_292_46_19044__index.html]

A unique cysteine-rich Zn-finger domain present in a majority of class II ribonucleotide reductases mediates catalytic turnover — A unique cysteine-rich zinc finger domain present in a majority of class II ribonucleotide reductases mediates catalytic turnover — Catalytic turnover in class II ribonucleotide reductase — Supplemental Data 

# A unique cysteine-rich zinc finger domain present in a majority of class II ribonucleotide reductases mediates catalytic turnover

## Supplemental Data

- Supporting information (.pdf, 59 KB) - Supporting information
- Supplementary Table S1 (.xlsx, 44 KB) - Supplementary Table
