# Supplementary material for: A unique cysteine-rich zinc finger domain present in a majority of class II ribonucleotide reductases mediates catalytic turnover
Source: J Biol Chem. 2017 Oct 2;292(46):19044–54. doi: 10.1074/jbc.M117.806331 (PMC5704485; doi:10.1074/jbc.M117.806331)
Supplement: Supplemental Data [file 10.1074_M117.806331_jbc.M117.806331-1.pdf]

Supplemental Data: A unique cysteine-rich Zn-finger domain present in a majority of class II ribonucleotide reductases mediates catalytic turnover

Christoph Loderer[‡], Venkateswara Rao Jonna[§], Mikael Crona[1#], Inna Rozman Grinberg[‡], Margareta Sahlin[‡], Anders Hofer[§], Daniel Lundin[‡*], Britt-Marie Sjöberg[‡*]

[‡]Department of Biochemistry and Biophysics, Stockholm University and [§]Department of Medical Biochemistry, Umeå University, Sweden
[#]current address: Swedish Orphan Biovitrum AB, Solna, Sweden
*corresponding authors:
Daniel Lundin, telephone: +46-8-52481520, fax: +46-8-155597, e-mail: daniel.lundin@dbb.su.se
Britt-Marie Sjöberg, telephone: +46-8-164150, fax: +46-8-155597, e-mail: britt-marie.sjoberg@dbb.su.se

Full phylogenetic trees

Complete tree files in nexml format (from Dendroscope) have been deposited to Figshare.
Full NrdJ tree: https://doi.org/10.17045/sthlmuni.5178415
Tree without NrdJm: https://doi.org/10.17045/sthlmuni.5178424

Sequence analysis matrix

Supplementary table S1. Analysis of sequence clusters. Representative sequences of NrdJ sequence clusters, excluding subclass NrdJm, with information about presence/absence of the CRD, subclass, presence of a separate gene encoding the CRD, cysteine pair in C-terminus (CRD or not) and alternative NrdJ with CRD elsewhere in genome.

The table is available as separate excel file: *Supplementary_Table_S1.xlsx*
